# Angiopoietin-like protein 8 (betatrophin) may inhibit hepatocellular carcinoma through suppressing of the Wnt signaling pathway 

**DOI:** 10.22038/ijbms.2019.36612.8764

**Published:** 2019-10

**Authors:** Nastaran Monzavi, Seyed Jalal Zargar, Nematollah Gheibi, Mahdi Azad, Babak Rahmani

**Affiliations:** 1 Department of Cell & Molecular Biology, School of Biology, College of Science, University of Tehran, Tehran, Iran; 2 Cellular & Molecular Research Center, Qazvin University of Medical Sciences, Qazvin, Iran; 3 Department of Biotechnology, School of Paramedical Sciences, Qazvin University of Medical Sciences, Qazvin, Iran

**Keywords:** ANGPTL8 protein, Beta-catenin, Carcinoma, Hepatocellular, Wnt inhibitory factor 1, Wnt pathway

## Abstract

**Objective(s):**

Hepatocellular carcinoma (HCC) is one of the leading fatal neoplasms and the most common primary liver malignancy worldwide. Peptide hormone ANGPTL8 (betatrophin) may act as an important regulator in HCC development through the Wnt/β-catenin pathway. We aimed to evaluate the effects of recombinant ANGPTL8 on Wnt/β-catenin signaling in human liver carcinoma cells (HepG2) and their viability.

**Materials and Methods:**

The expression of ANGPTL8 was conducted in the pET-21b-E. coli Bl21 (DE3) system and the produced peptide was purified. HepG2 cells were treated with different concentrations of ANGPTL8 (25, 50, 100, 150, 200, and 250 ng/ml) for 24, 48, and 72 hr. MTT assay was performed to detect the viability of treated cells, and apoptotic induction by ANGPTL8 was measured by flow cytometry assay. Finally, using qRT-PCR the mRNA levels of Wnt signaling modulators WIF-1 and β-catenin were evaluated in treated cells.

**Results:**

MTT assay showed that ANGPTL8 inhibits proliferation of HepG2 cells moderately in a time-independent manner. The highest concentration of the ANGPTL8, 250 ng/ml, reduced cell proliferation after 24, 48, and 72 hr similarly about 30%. In the same concentration of ANGPTL8, after 24 hr of treatment flow cytometry assay revealed a mild increase in early and late apoptosis up to 7.7 and 14.3%, respectively. The qRT-PCR showed that in a concentration-dependent and time-independent fashion, the expression of WIF-1 and β-catenin genes respectively increased and decreased significantly (*P*<0.05).

**Conclusion:**

Our findings suggest that ANGPTL8 may act as a moderate suppressor against HCC cell proliferation possibly via affecting Wnt signaling modulators.

## Introduction

Hepatocellular carcinoma (HCC) is ranked the sixth common cancer worldwide and the second leading cause of cancer-related deaths (1). HCC patients are usually diagnosed at late stages, and comparing to other common malignant tumors its prognosis is poor (2). Since limited treatment options for patients with advanced HCC exist, it has become a challenge to find novel therapies for this global health problem (3). So, looking for causative factors and studying them seems to be helpful in searching for more efficient therapies. Major risk factors for HCC incidence include viral hepatitis, cirrhosis (4), and metabolic disorders (5). Indeed, it is predicted that metabolic syndrome (MtS) could result in a large increase in the incidence of HCC in the future (6). Therefore, studying the molecular players of this disorder, which may contribute to HCC induction or suppression is an open research topic waiting to be investigated more meticulously.

Angiopoietin-like protein 8 (ANGPTL8) with alternative names such as C19orf80, RIFL, lipasin, ANGPTL8, TD26, and LOC55908 is a secretory protein, which is expressed predominantly in the human liver. The corresponding gene is located on chromosome 19p13.2 and its transcript contains four exons, which encodes a protein composed of 198 amino acids (7, 8). Recently, ANGPTL8 was introduced as an emerging player in lipid metabolism and homeostasis of metabolic events (9). For example, it binds to lipoprotein lipase and regulates triglyceride metabolism (10). Also, alteration of ANGPTL8 concentration in metabolic diseases including diabetes, obesity, and MtS has been revealed by many epidemiological studies. These findings indicate that ANGPTL8 may play a role in the emergence and progression of many diseases (11). In the case of HCC, the mRNA expression profile of ANGPTL8 is concordant with the α-fetoprotein gene, which is an HCC-associated gene and diagnostic and prognostic biomarker of this cancer in several liver cancer cell lines (12). Further, it is shown that the ANGPTL8 locus is hypermethylated in cirrhotic liver compared with normal liver and this hypermethylation is also maintained in HCC (13). However, in a study, ANGPTL8 was reported among genes that are overexpressed in HCC (14).

One of the main involved molecular pathways in HCC is the Wnt/β-catenin pathway. This signaling pathway is frequently upregulated in HCC and maintains tumor-initiating cells and involves in drug resistance, tumor progression, and metastasis of HCC cells (15). Dysregulation of the Wnt signaling cascade has been observed in approximately 95% of HCC cases and components of the pathway represent a different expression profile (16). For instance, one of the regulators of the pathway, β-catenin, accumulates in HCC tissues in an association with decreased expression of the other Wnt signaling pathway component DKK4 (17). Also, down-regulation or missing expression of Wnt inhibitory factor 1 (WIF-1) is reported in HCC cell lines. Therefore, up-regulation of WIF-1 expression was proposed as a significant inhibiting way against invasion and metastasis of HCC cells (18). Consequently, any substance, which may equilibrate this pathway and result in HCC suppression could be valuable for further studies in anti-hepatocarcinoma research lines. Additionally, it is noteworthy that Wnt signaling has molecular acts in lipid metabolism in hepatocytes and hepatocarcinoma cells (19). It is reported that reducing β-catenin expression decreases expression of enzymes, which are involved in fatty acid esterification in hepatic lipid metabolism (20), and it may suggest a link between the Wnt pathway and ANGPTL8 protein.

Paying attention to the increasing effect of MtS on HCC incidence and possibility of MtS factors contribution in this cancer, also, considering the Wnt signaling pathway involvement in HCC and liver lipid metabolism, and regulatory function of ANGPTL8 in the lipid metabolism and metabolic disorders, this study was conducted to investigate the possible effect of ANGPTL8 protein on the proliferation of the HCC cell line HepG2 and on the expression levels of two important Wnt signaling regulators β-catenin and WIF-1 in these cells.

## Materials and Methods

***Expression and purification of ANGPTL8***

At first, *E. coli* BL21 bacteria, which contain the recombinant plasmid pET21-ANGPTL8 (21) were cultured using the streak-plate technique to obtain a single colony. Then, the colony was inoculated in a 5 ml LB broth containing 50 µg/ml ampicillin and incubated at 37 ^°^C overnight. After that, 50 ml of the LB broth was inoculated with 0.5 ml of the overnight culture. Ten micrometers of the inducer (IPTG) was added after it attained an OD600nm of 0.6 and incubated for 6 hr. To determine the optimized time and concentration of induction, the test was repeated several times. The expressed protein was purified using Ni-NTA agarose column following the manufacturer’s instructions (Qiagen, Hilden, Germany). The purified protein was analyzed on a 12% SDS-PAGE gel electrophoresis, dialyzed and refolded with PBS at 4 ^°^C overnight.

**Figure 1:**
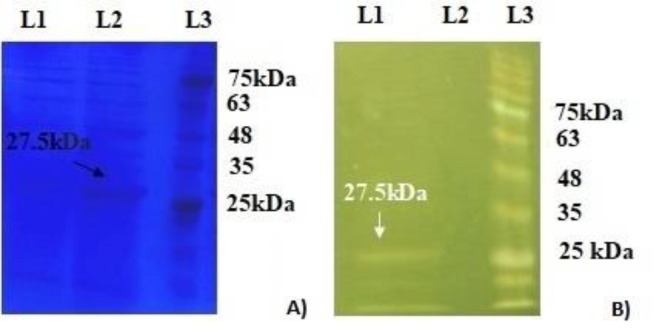
SDS-PAGE of expressed recombinant ANGPTL8. Lane1: uninduced bacteria, Lane2: induced bacteria, Lane3: protein marker (A). Purification of recombinant ANGPTL8 was performed using Ni-IDA chromatography, which is demonstrated in Lane1: purified protein; Lane2: supernatant, Lane3: protein marker (B)

**Figure 2:**
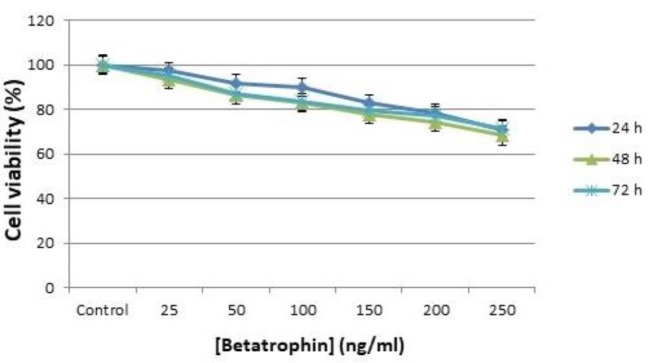
Cell viability assay using MTT test. Effect of different concentrations of ANGPTL8 on the viability of HepG2 cells after 24, 48, and 72 hr treatment with peptide hormone compared with the control

**Figure 3:**
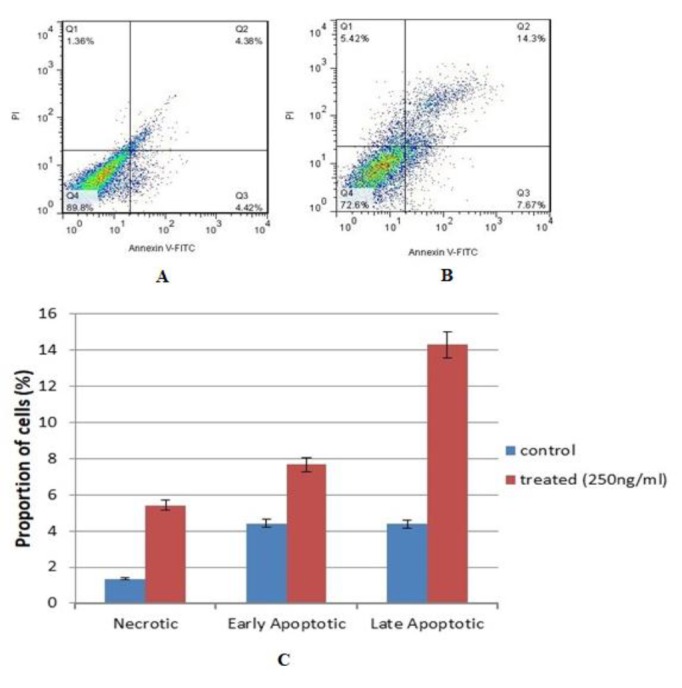
Flow cytometric analysis of ANGPTL8 (250 ng/ml) induced apoptosis in HepG2 cells using FITC-conjugated Annexin V and PI. Untreated (control) cells after 24 hr (Annexin V-, PI-) (A), Treated cells with ANGPTL8 after 24 Figure 4. Effect of different concentrations of ANGPTL8 on the expression level of WIF-1 and β-catenin in HepG2 cells. In 24 (A), 48 (B), and 72 hr (C) after treatment, exposure of cells to the increased levels of ANGPTL8 led to respectively increased and decreased expression of WIF-1 and β-catenin. This expression change was time-independent, however, it was influenced by the concentration of recombinant peptide hormone *; *P*<0.05, #; *P*<0.05 hr (B), The percentages of necrotic cells (Annexin V-, PI+), early apoptotic cells (Annexin V+, PI-), and late apoptotic cells (Annexin V+, PI+) (C)

**Figure 4:**
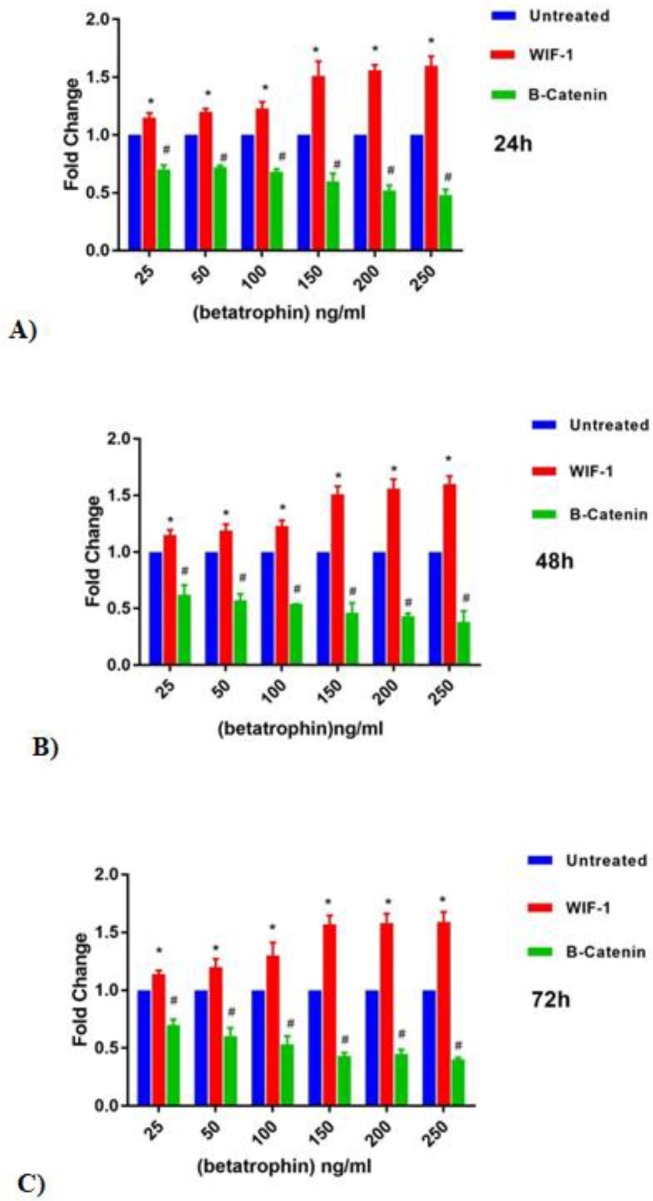
Effect of different concentrations of ANGPTL8 on the expression level of WIF-1 and β-catenin in HepG2 cells. In 24 (A), 48 (B), and 72 hr (C) after treatment, exposure of cells to the increased levels of ANGPTL8 led to respectively increased and decreased expression of WIF-1 and β-catenin. This expression change was time-independent, however, it was influenced by the concentration of recombinant peptide hormone *;* P*<0.05, #; *P*<0.05

**Table 1:** The sequences of primers used in this study

**Gene**	**Forward 5’ to 3’**	**Reverse 5’ to 3’**
β-catenin	5’ -CTCTGATAAAGGCTACTGTTGGATTGATTC -3’	5’-TTGCTGCTGTGTCCCACCCA -3’
WIF-1	5′ - GCACGGCAGACACTGCAATAA-3′	5’-AAAGGCTGTGAACCCGGTGTAA-3’
GAPDH	5’-CAATGACCCCTTCATTGACC-3’	5’-TGGAAGATGGTGATGGGATT-3’

***Cell culture***

The HepG2 cell line (NCBI Code: C158) was obtained from the National Cell Bank of Iran (NCBI, Pasteur Institute of Iran, Tehran). It was grown at 37 °C under an atmosphere composed of 5% CO2 and 95% air in a cell culture flask. Ten milliliters of Dulbecco’s Modified Eagle’s Medium containing 10% fetal calf serum, 1% L-glutamine, 1% penicillin, and 1% streptomycin was used. Passaging was done via trypsinization when the cells reached 80% of confluency. 

***Cytotoxicity assay***

Cell viability was tested using the MTT assay, which is based on the cleavage of the tetrazolium salt (MTT) by metabolically active cells to form a water-insoluble formazan dye. The HepG2 cells were seeded in 96-well plates at a concentration of 5×10^3^ cells/well and incubated for 24 hr. After that, the media was discarded and the cells were exposed to various concentrations of ANGPTL8 (25, 50, 100, 150, 200, and 250 ng/ml of media) for 24, 48, and 72 hr. Then, the medium was removed and MTT was added to each well and incubated for 4 hr at 37 °C in the dark. After incubation, MTT was removed and replaced with 100 μl of DMSO and finally, the optical absorbance was measured at 570 nm with an ELISA plate reader. All experiments were performed in triplicate.

***Apoptosis assay by flow cytometry***

The apoptosis incidence in cells was investigated by flow cytometry using the Annexin V-FITC apoptosis detection kit (Biolegend). Generally, apoptosis starts with the translocation of phosphatidylserine from the inner part to the outer part of the plasma membrane. The binding of Annexin V to phosphatidylserines results in emission of green fluorescence. During late apoptosis, permeability increases and leads to the entry of Propidium Iodide (PI) into the cells, which upon binding to DNA changes the nucleus dye to red. At first, HepG2 cells were seeded in 6-well plates with a density of 5×10^5^ cells/well and were grown to 80% confluency. Then, they were treated with 250 ng/ml of ANGPTL8 for 24 hr. The cells were trypsinized and collected by centrifugation (350 g, 5 min) and were stained with Annexin V and PI and subjected to flow cytometric analysis using a BD FACSCalibur flow cytometer (BD Biosciences, USA).

***RNA extraction and Real-time PCR***

Total cellular RNAs of treated cells with different concentrations of ANGPTL8 and control cells were extracted using the High Pure Isolation Kit (Roche, Germany), following the manufacturer’s guidelines. Next, the extracted RNA was evaluated using a NanoDrop spectrophotometer at wavelengths of 260 and 280 nm to determine its quantity and quality. The extracted RNA was reverse transcribed to cDNA using the Roche kit, according to the manufacturer’s protocol. After that, real-time PCR experiments were done using an ABI StepOne qRT-PCR system, in order to detect the changes in the expression level of genes of interest. For this purpose, specific primers designed for the studied genes were used (Table 1). Glyceraldehyde-3-phosphate dehydrogenase (GAPDH) gene expression was considered as control. The qPCR amplification was conducted in 20 μl reaction buffer by SYBR Green PCR Master Mix under the following conditions: initial denaturation at 95 ^°^C for 5 min, 40 cycles at 95 ^°^C for 20 sec, annealing at 58 ^°^C for 20 sec, and extension at 72 ^°^C for 40 sec. All samples were run in triplicate. Data were analyzed using the Pfaffl method and expressed as E^-ΔΔCT ^(where E is the reaction efficiency). The graph was drawn by GraphPad Prism v6 software.

## Results

***Recombinant ANGPTL8 expression and purification***

The presence of a protein band about 27.5 kDa in induced bacteria was proven through SDS-PAGE analysis of bacterial homogenate (Figure 1A). The recombinant human ANGPTL8 protein was purified by Ni-NTA chromatography and the purification yield was 1.4 mg/ml. Again, SDS-PAGE analysis of isolated protein showed a band about 27.5 kDa (Figure 1B).

***Viability assay after treatment of cells with ANGPTL8***

The toxicity effect of ANGPTL8 on HepG2 cancer cells was time independent and by increasing the concentration of ANGPTL8 the viability of the cells was reduced slightly. As depicted in Figure 2 for the highest concentration of the recombinant peptide hormone, 250 ng/ml, cell proliferation reduced about 30% in the same fashion after 24, 48, and 72 hr.

***Induction of apoptosis***

To determine the apoptosis rates of HepG2 cells after treatment with ANGPTL8 at a concentration of 250 ng/ml, the flow cytometry technique was used. The obtained results are shown in Figure 3. ANGPTL8 increased the early and late apoptosis up to 7.7 and 14.3%, respectively.

***Expression changes of WIF-1 and β-catenin***

The expression of WIF-1 and β-catenin in cells treated with different concentrations of ANGPTL8 (25, 50, 100, 150, 200, and 250 ng/ml) for 24, 48, and 72 hr and the untreated control cells were compared. Figure 4 illustrates the expression ratio after standardization (fold change) of the WIF-1 and β-catenin genes. Treatments with same concentrations in different times resulted in approximately the same changes in the gene expression. However, expression changes were concentration-dependent as described in Figure 4.

## Discussion

Like other cancers, hepatocellular carcinoma is a polygenic disease with complex mechanisms and several dysfunctions in signaling pathways (22). This molecular ambiguity puts many obstacles in the way of researchers to achieve a successful therapeutic discipline for HCC (23). However, biotechnological approaches including immunotherapy (24) and molecular targeted therapies (25) like the silencing of key player genes (26) are emerging opportunities as important treatment options in this regard.

One of the aberrated pathways in HCC is Wnt/β-catenin signaling. Especially, its canonical pathway is upregulated in approximately 95% of observed cases and most of the pathway ligands and their receptors have been shown to be overexpressed in HCC cells. So, as a central player in maintaining liver health, this pathway provides a promising target for molecular therapies of HCC (16, 27, 28). Yang *et al.* showed that Wnt/β-Catenin signaling activates normal and tumorigenic liver progenitor cells. They believe that Wnt-pathway targeting therapies may provide a specific method to disrupt the chemoresistance mechanism of hepatocarcinoma cells (29). Two of the key regulatory molecules involved in this pathway are WIF-1 and β-catenin (30). WIF-1 is a secreted antagonist capable of direct binding to Wnt proteins in the extracellular space to inhibit the Wnt pathway (31). The subnormal function of WIF-1 can result in carcinogenesis through the Wnt pathway overactivation and leads to dysregulation of cell proliferation and differentiation (32). Indeed, WIF-1 down-regulation in tumors including HCC has been reported by several studies previously (28, 31). Also, cytoplasmic and nuclear accumulation of another key regulator of the pathway, β-catenin, has been nearly demonstrated in 40–70% of HCC cases. Indeed, canonical pathway way activation is dependent on β-catenin function (28, 33). It is shown by classic studies that somatic mutations in the β-catenin gene activate the Wnt/β-catenin pathway in HCC, which may stimulate tumor cell proliferation and promote tumor progression (34, 35).

One of the newly recognized important risk factors of HCC incidence is MtS. It seems that biochemical alterations induced by MtS could result in an increase of the HCC malignancy (5). Related to the molecular regulating of MtS, ANGPTL8 protein was introduced as an emerging player recently. It has an important function in lipid metabolism and its concentration is altered in MtS cases (9, 11). Surprisingly, ANGPTL8 has some molecular interactions with HCC (12, 14) and liver cirrhosis (13) and it has been shown that its expression could be induced by different inflammatory stimuli in HepG2 cells (36). Besides that, the Wnt signaling pathway has substantial interaction with lipid metabolism pathways. For instance, Wnt proteins are lipid-modified (37) and molecules such as palmitoleic acids are involved in these processes (38). Considering the ambiguity of these molecular connections, we aimed to study the possible effects of recombinant ANGPTL8 on the Wnt/β-catenin pathway in HCC using the HepG2 cell line. HepG2 cells contain a truncation mutation in the β-catenin gene, which results in removal of the encoded protein’s key regulatory sites. This event causes the aberrant accumulation of β-catenin for constitutive activation of the Wnt/β-catenin pathway in HepG2 cells (39, 40).

To achieve this goal, we investigated cell proliferation and expression level of the two main Wnt pathway regulator genes, WIF-1 and β-catenin, under ANGPTL8 treatment. The production of recombinant ANGPTL8 in our cloning system was proven through SDS-PAGE analysis and the corresponding band was absent in un-induced bacteria (Figure 1A). Apparently, the molecular weight of recombinant ANGPTL8 was about 27.5kDa, which is higher than the estimated molecular mass of human ANGPTL8 protein with 22 kDa weight. This happened due to the presence of the 6-His tag with 2.7 kDa weight plus 2.8 kDa of other plasmid-encoded amino acids. It is worth noting that we exactly followed the recombination procedure as described by Gholami *et al.* and they demonstrated that their protocol resulted in a purified recombinant ANGPTL8 by the same secondary structure, which original ANGPTL8 has (21). The treatment results showed that ANGPTL8 decreased cell proliferation up to 30% and this reduction was dependent on concentration increase and independent of time. In addition, in comparison with the control cells, the evaluation of apoptosis in treated HepG2 cells by annexin V-FITC showed nearly 13.2% increase of early and late apoptosis altogether. Therefore, ANGPTL8 showed a moderate inhibitory effect on the growth of HepG2 cells.

Meanwhile, the real-time PCR results demonstrated that the expressions of β-catenin and WIF-1 in treated cells were changed. Figure 4 illustrates the expression ratios after standardization (fold change) of the WIF-1 and β-catenin genes expression. In contrast to the control, in the treated samples gene expression for β-catenin and WIF-1 was reduced and increased respectively and these changes depended only on ANGPTL8 concentration but not on treatment time courses. It shows that induction of ANGPTL8 expression in HCC cells may revise the altered pattern of the Wnt signaling regulators expression and elevate the decreased level of WIF-1 and down-regulate upraised level of β-catenin. The study results suggested that induction of ANGPTL8 protein may have a moderate anti-hepatocarcinoma effect via balancing of Wnt signaling regulators, however, its action mechanisms are still unknown and further detailed cellular and molecular studies especially on the protein network connection between HCC, MtS factors, and Wnt signaling pathway are necessary. 

## Conclusion

Overall, the present study revealed that ANGPTL8 affects mRNA expression of two key regulators of Wnt signaling pathway, β-catenin and WIF-1, and inhibits proliferation of the hepatocellular carcinoma cell line HepG2 moderately. So, ANGPTL8 may act as a mild suppressor against hepatocellular carcinoma cells. However, more detailed studies must be conducted to unravel the exact molecular aspects of ANGPTL8 role in cancer pathways. 
